# Characteristics recurrence pattern of cholangiolocellular carcinoma as intrahepatic bile duct tumor growth following curative resection: a case report

**DOI:** 10.1186/s40792-019-0698-2

**Published:** 2019-09-05

**Authors:** Keishi Hakoda, Tomoyuki Abe, Hironobu Amano, Tomoyuki Minami, Tsuyoshi Kobayashi, Keiji Hanada, Kenji Nishida, Shuji Yonehara, Masahiro Nakahara, Hideki Ohdan, Toshio Noriyuki

**Affiliations:** 1 Department of Surgery, Onomichi General Hospital, 23-10-1 Hirahara, Onomichi City, Hiroshima, 7228508 Japan; 2 Department of Gastroenterological and Transplant Surgery, Applied Life Sciences, Institute of Biomedical & Health Sciences, Hiroshima University, Hiroshima, Japan; 3 Department of Gastroenterology, Onomichi General Hospital, Onomichi, Japan; 4 Department of Pathology, Onomichi General Hospital, Onomichi, Japan

**Keywords:** Cholangiolocellular carcinoma, Neoplasm local recurrence, Hepatectomy

## Abstract

**Background:**

Cholangiolocellular carcinoma (CoCC) is a rare primary liver tumor that shows mass-forming growth in most cases. At present, no effective treatment for hepatic recurrence CoCC has been established. We present a case involving a patient with recurrent disease that showed an intraductal growth (IG type) pattern of recurrence. The patient was treated with repeat hepatectomy with bile duct reconstruction.

**Case presentation:**

The patient was a 76-year-old man with a history of S8 subsegmentectomy for CoCC. At 8 months after surgery, tumor marker elevation was observed. Computed tomography revealed a tumor occupying the right hepatic duct (B5-8) to B4 and the junction of the cystic duct. Endoscopic retrograde cholangiopancreatography (ERCP) and a thrombus biopsy with peroral cholangioscopy (POCS) confirmed the recurrence of CoCC in the intrahepatic bile duct. Although extended right lobectomy with extrahepatic bile duct resection was the optimal curative procedure, it was thought that it would be difficult due to his poor liver function. However, a slow-glowing recurrent tumor blocked the posterior branch of the portal vein; thus, the right liver lobe gradually shrank, and the estimated remnant liver volume increased in response, allowing curative surgery to finally be performed. At 10 months after surgery, the patient is alive without recurrence.

**Conclusions:**

We reported a case of IG-type recurrence in the bile duct, which is an unusual pattern of intrahepatic recurrence, after initial surgery for CoCC. A slow-growing recurrent tumor exerted similar effects to PVE, which allowed for curative surgery to be performed.

## Background

Cholangiolocellular carcinoma (CoCC) is a rare liver tumor that generally shows mass-forming (MF)-type growth [1–3]. It is reported that patients with CoCC show favorable survival outcomes after curative resection [4], with the most frequent site of recurrence being the liver [5]. The efficacy of repeat hepatectomy for hepatocellular carcinoma (HCC) and intrahepatic cholangiocarcinoma (ICC) has been proven [6, 7]; however, the efficacy of this approach in CoCC is unclear.

We herein report the case of a patient with intraductal growth (IG)-type recurrence of CoCC who underwent extended right lobectomy with extrahepatic bile duct resection.

## Case presentation

**Fig. 1:**
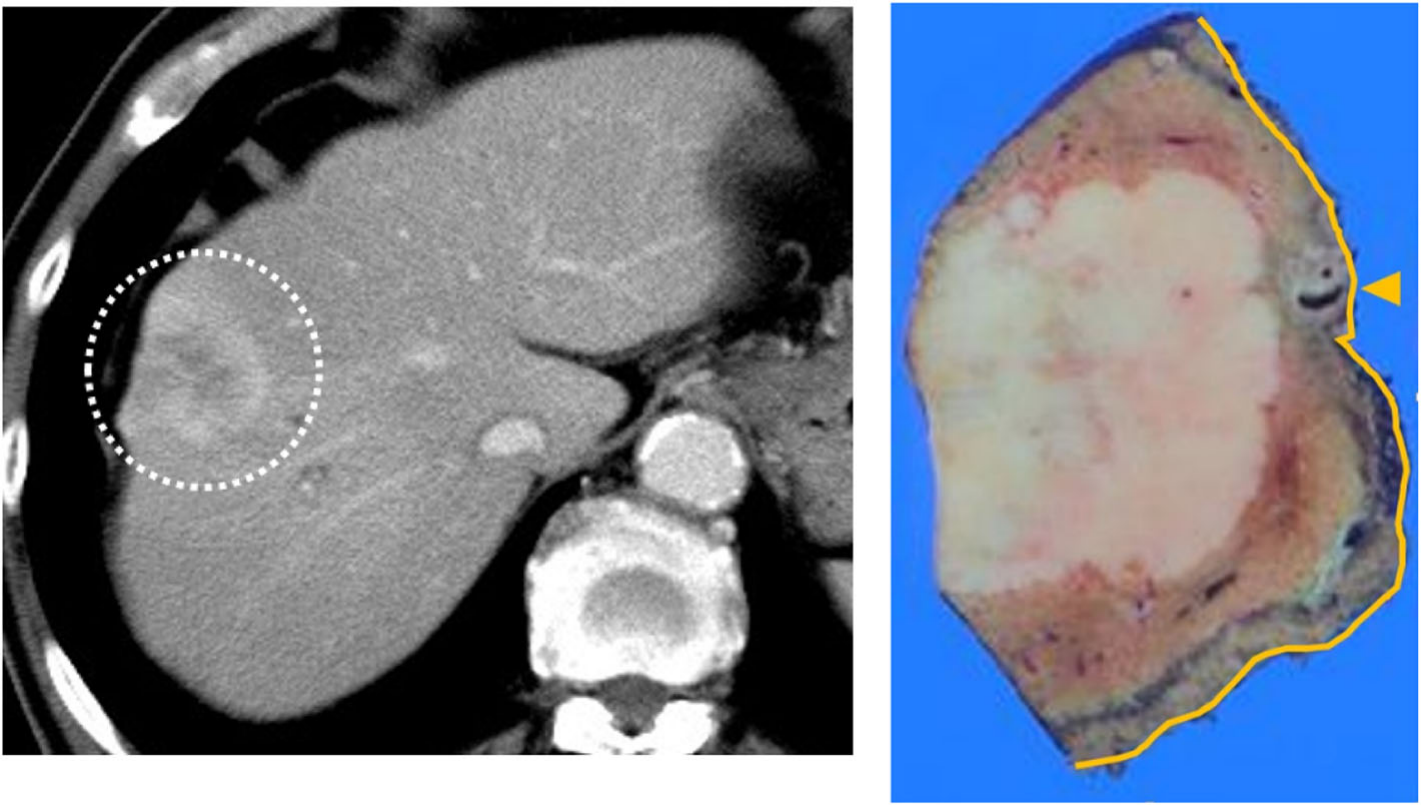
Computed tomography (axial image, white-dotted line circle) revealed an enhanced tumor. A macroscopic examination of the primary tumor revealed mass-forming type growth. The S8 Glisson stump (yellow arrow) and hepatic radial margin (yellow line) were tumor-free

The patient was a 76-year-old man. A laboratory analysis was positive for hepatitis B surface antigen. He had a medical history of S8 subsegmentectomy for CoCC. According to the Child–Pugh classification, his liver function was stage B and he had grade B liver damage. A macroscopic examination revealed an MF-type tumor. The final diagnosis was T2N0M0 (size 35 × 37 mm, im[-], eg, fc[-], sf[-], s0, vp0, vv0, va0, b1, sm[-]) (Fig. 1). Most of the tumor consisted of the area of CoCC with peripheral intrahepatic bile duct infiltration (b1) in the vicinity of the hepatic radial margin.

**Fig. 2:**
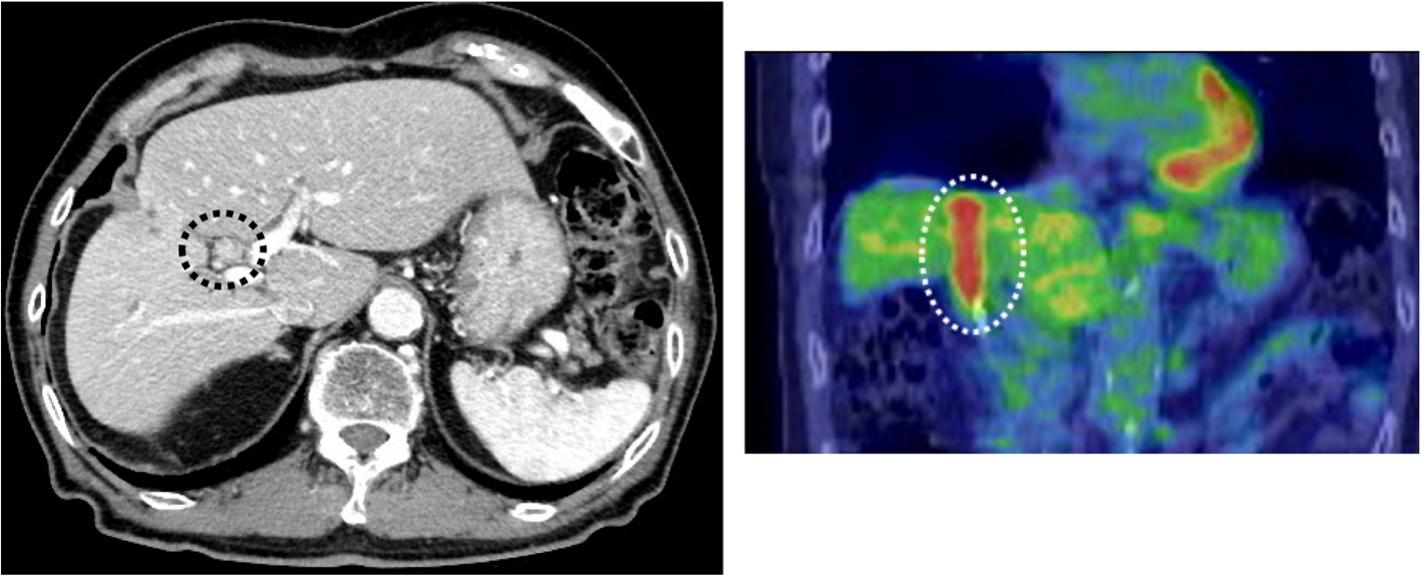
Computed tomography (axial image, black-dotted line circle) and PET–CT (coronal image, white-dotted line circle) revealed an intrahepatic bile duct tumor with the accumulation of FDG

**Fig. 3:**
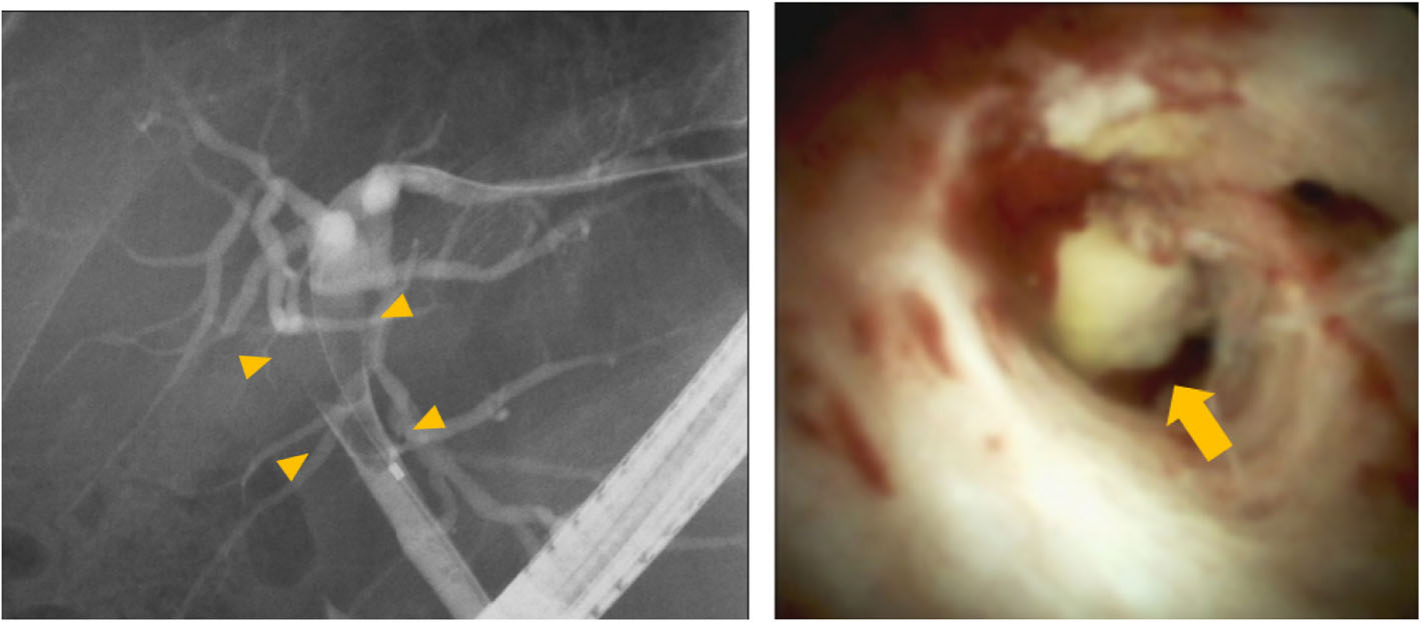
Endoscopic retrograde cholangiopancreatography (ERCP) showed intrahepatic duct tumor thrombus within the right hepatic duct (B5-8) and B4 to the junction of the cystic duct (arrowhead). The tumor was observed by peroral cholangioscopy (POCS) (arrow)

At 8 months after surgery, tumor marker elevation was observed (CA19-9, 309 U/ml). His liver function was classified as Child–Pugh stage B, and the grade of liver damage was classified as grade B. Positron emission tomography–computed tomography (PET-CT) revealed an enhanced tumor occupying the common bile duct to the right hepatic bile duct with the accumulation of fluorodeoxyglucose (Fig. 2). Endoscopic retrograde cholangiopancreatography (ERCP) showed intrahepatic bile duct tumor thrombus within the right hepatic bile duct (B5-8) and B4 to the junction of the cystic duct. A tumor biopsy through peroral cholangioscopy (POCS) revealed similar findings to the primary tumor; thus, the diagnosis was recurrent CoCC, which displayed IG type growth pattern (Fig. 3).

**Fig. 4:**
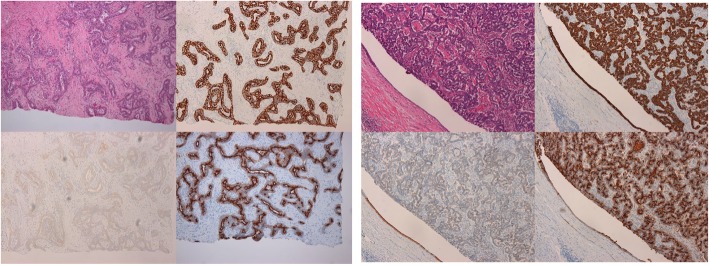
The histopathological findings of the primary and recurrent tumors (upper left). Immunohistochemistry of both tumors showed the positive expression of cytokeratin (CK) 7, CK19, and EMA (lower left, upper right and lower right)

The histopathological findings of the primary tumor and recurrent tumor both revealed that the proliferating tumor cells were composed of antler-like and anastomosing patterns with abundant fibrous stroma. Immunohistochemistry revealed the positive expression of cytokeratin (CK) 7, CK19, and epithelial membrane antigen (EMA). EMA was also positive on the luminal surfaces of the tubules (Fig. 4). Extended right lobectomy with extrahepatic bile duct resection was the only curative surgical procedure; however, at the time of the initial diagnosis, it was deemed difficult due to his poor liver function and because the expected remnant liver volume was insufficient. However, while discussing further treatment, a slow-glowing recurrent tumor blocked the posterior branch of the portal vein, causing the right liver lobe to gradually shrink, and the remnant liver volume and ICG Krem ultimately increased to 52% and 0.058, respectively [8]. At 1 year after the initial surgery, extended right lobectomy with extrahepatic bile duct resection was performed. The only postoperative complication was ascites (Clavien–Dindo grade II), which was controlled with tolvaptan. A pathological examination indicated recurrent CoCC forming intrahepatic bile duct thrombus; the Ki-67 expression of the tumor was high (14.5%). The recurrent tumor had slightly invaded a bile duct, while the right portal vein had not been invaded. The surgical margin was sufficient. The patient remains alive without recurrence at 10 months after the second hepatectomy.

## Discussion

CoCC is a rare liver tumor accounting for 0.6–1% of primary liver tumors and was first reported by Steiner and Higginson in 1959 [1, 2]. CoCC is derived from the canals of Hering, or cholangioles, where hepatic progenitor cells are located [9]. A previous report describing the pathological features of 20 cases CoCC noted that macroscopically, all cases were of the MF type [3]. Curative hepatectomy for CoCC is reported to be associated with a higher 5-year survival rate (75%) in comparison to ICC, with liver metastasis being the most frequent pattern of recurrence (75%) [5]. There is currently no established therapy for hepatic recurrence of CoCC; however, repeat hepatectomy has been reported to be an effective treatment for patients with limited hepatic recurrence of HCC and ICC [6, 7]. Given that repeat hepatectomy has been reported to be associated with good long-term outcomes in some cases involving hepatic recurrence of CoCC [10, 11], aggressive surgical resection is considered to be an effective treatment, as it is for other liver tumors. It is also reported that surgical resection for intraductal growth (IG type) recurrence of HCC and ICC prolongs the survival time of patients [10, 12]. Considering the relatively low malignant potential of CoCC, aggressive surgical treatment for IG type recurrence may also be acceptable. However, at present, there is no established strategy for managing recurrent CoCC; thus, systemic chemotherapy and radiation treatment can be applied in cases of distant recurrence. Further investigations are necessary to determine the validity of this approach.

Given the low liver function of the present patient at the time of the first operation, we considered that he would not be able to tolerate both right lobectomy and anterior segmentectomy; thus, S8 segmentectomy was performed as the only feasible surgical procedure. As a result, IG-type recurrence developed 8 months after the first surgery. Considering his remnant liver function, we deemed further surgery difficult; however, a slow-growing recurrent tumor gradually blocked the posterior branch of the portal vein and subsequently reduced the blood flow, causing the right liver lobe volume to shrink while the remnant left liver lobe volume increased. The clinical course in this case was similar to that observed in patients after portal vein embolization (PVE). PVE is a procedure in which the portal vein in the part of the liver that is to be resected is embolized in advance of a surgery to induce the regrowth of the remnant liver. This procedure allows the patient to have a sufficient liver function after the operation.

The macroscopic growth pattern of the tumor differed between the two operations. The former pattern was MF type, whereas the latter was IG type. The reason for this change in macroscopic features was unclear; however, the primary tumor infiltrated the peripheral intrahepatic bile duct (b1) in the vicinity of the hepatic radial margin, and immunohistochemistry of the recurrent tumor revealed the high expression of Ki-67 (14.5%). These findings may have contributed to the change in the growth pattern and the relatively early recurrence.

We used the remnant liver volume and ICG Krem value as indices to indicate the performance of extended right lobectomy with bile duct resection. The remnant liver volume could be calculated using multi-detector row helical CT and was determined to be 52% in this case. ICG Krem is determined by multiplying the disappearance rate of indocyanine green by the ratio of the remnant liver volume to the total liver volume. In patients with a low liver function, a preoperative ICG Krem value of ≥ 0.05 indicates that major hepatectomy can be safely performed [8]. In the present case, the patient’s ICG Krem value was 0.058. The only postoperative complication was ascites (Clavien–Dindo grade II), which suggests that both remnant liver volumetry and the calculation of the ICG Krem value were useful for assessing the risk of postoperative liver failure.

## Conclusion

We reported the case of a patient with recurrent CoCC located in the intrahepatic bile duct with an IG type growth pattern, which is an unusual pattern of recurrence. A slow-growing recurrent tumor exerted similar effects to PVE, resulting in an increase in the remnant liver volume, which allowed extended right lobectomy with extrahepatic bile duct resection to be performed.

## Data Availability

Data sharing is not applicable to this article as no datasets were generated or analyzed during the current study.

## Abbreviations

CK: Cytokeratin
CoCC: Cholangiolocellular carcinoma
EMA: Epithelial membrane antigen
ERCP: Endoscopic retrograde cholangiopancreatography
HCC: Hepatocellular carcinoma
ICC: Intrahepatic cholangiocarcinoma
IG: Intraductal growth
MF: Mass-forming
PET-CT: Positron emission tomography–computed tomography
POCS: Peroral cholangioscopy
PVE: Portal vein embolization
